# Research on the Construction and Prediction of China's National Fitness Development Index System Under Social Reform

**DOI:** 10.3389/fpubh.2022.878515

**Published:** 2022-05-16

**Authors:** Zheng Liu, Shijia Zhang, Lingling Li, Bin Hu, Ran Liu, Zhao Zhao, Yuanjun Zhao

**Affiliations:** ^1^School of Management, Shanghai University of Engineering Science, Shanghai, China; ^2^Department of Central Laboratory, Shanghai Children's Hospital, Shanghai Jiao Tong University, Shanghai, China; ^3^School of Economics and Management, Tongji University, Shanghai, China; ^4^Odette School of Business, University of Windsor, Windsor, ON, Canada; ^5^School of Accounting, Nanjing Audit University, Nanjing, China; ^6^Institute of Intelligent Management Accounting and Internal Control, Nanjing Audit University, Nanjing, China

**Keywords:** national fitness, evaluation index system, balanced scorecard, social reform, prediction

## Abstract

**Background:**

National fitness is a development plan formulated by China to promote people's participation in leisure and fitness, enhance people's physique, and realize the general goal of strengthening sports.

**Methods:**

Based on combing the development process of China's national fitness after reform and opening up, using the improved “balanced scorecard” method, this article constructs an evaluation index system of the national fitness development index.

**Results:**

The national fitness development index was established, including 4 first-level indicators, 14 second-level indicators, and 49 third-level indicators. It can calculate the national fitness development index with a total score of 100 points.

**Conclusion:**

To verify the feasibility of the evaluation system, the goal current situation evaluation method is used to calculate the national fitness development index during the 14th Five Year Plan period based on the development of national fitness during the 13th Five Year Plan period to provide evaluation tools and theoretical reference for the development of national fitness in China.

## Introduction

On October 20, 2014, The State Council of China issued several opinions on accelerating the development of the sports industry and promoting sports consumption, which clearly stated: “We must develop mass sports in an all-round way, constantly meet the growing demand of the people for sports, and make national fitness a national strategy (1).” The national fitness development of China is so far from the in-depth development stage into the optimization and upgrading stage (2). National fitness is a development plan formulated by China to promote leisure and fitness participation, enhance people's physique, improve people's health level, and realize the general goal of building a strong sports country (3). After years of efforts, the national fitness strategy has been deeply implemented, the level of public services has been significantly improved, and the proportion of people who often participate in physical exercise has reached 37.2%. At the same time, there are still some problems in the development of national fitness in China, such as the structural imbalance between supply and demand of public services, the insufficient distribution of the role of the market mechanism, and the insufficient construction of standardization (4).

Against the historical background of the new era, China is at a historical moment of moving from a great sports country to a great sports country (5, 6). With “Healthy China 2030” as the blueprint, the national fitness campaign is widely promoted to improve the health of the whole people (7). The Chinese government regards the national fitness strategy as a sports industrial revolution and adopts a series of effective measures to develop the sports environment and improve the participation rate of national fitness (8). Public service facilities are the physical space of national fitness and constructing public sports facilities is the key content of national fitness (9). The research shows that more than half of the residents are satisfied with the current national fitness public service, but problems such as low standardization, unequalization of service, and lack of professional talents still exist (10). Equalization of national fitness public services is to ensure that all citizens enjoy equal opportunities for fitness services (11). With the improvement of people's living standards, the contradiction between people's growing demand for sports service and the relatively backward sports service is becoming increasingly prominent (12). The goal of “national fitness and urban integration” can coordinate the layout of urban and rural public sports resources and services and achieve the equalization of basic public services of national fitness for residents (13). Community is the basic carrier of urban public services, so we should strengthen the construction of fitness facilities in urban communities, improve the fitness literacy of drama titles, and integrate sports activities into people's daily life (14). The construction of a policy system focusing on national fitness can rely on the driving force behind the policy to promote the progress of national fitness in organizational structure, laws and regulations, personnel training, and other aspects. The research shows that there is an asymmetric mutualistic symbiotic relationship between national fitness and the sports industry for a long time (15). In developing countries, sports have made a significant contribution to improving people's wellbeing (16).

The construction of the evaluation system of national fitness, scholars from different dimensions is studied in “sports power construction program” as the basic framework, build the mass sports, competitive sports, sports industry, sports culture, sports diplomacy, security system, and negative indicators of seven dimensions in a new era of comprehensive evaluation index system of sports development (17). The mass sports evaluation index system takes the goal realization, the main aspect, the development foundation, and the support system as the system-level evaluation index (18). The local government national fitness public service performance evaluation system is composed of four dimensions: efficiency, quality, democracy, and responsiveness. Ningbo national fitness basic public service quality evaluation system is composed of public sports facilities quality, public sports service quality, fitness environment quality, affiliated sports facilities quality, and mass fitness service personnel quality (19).

The development of national fitness is related to the effective implementation of “sports power strategy” and “healthy China strategy.” Existing studies lack a systematic and holistic development index evaluation system for national fitness, leading to the inability to quantify the development of national fitness, which is not conducive to scientific decision-making by government agencies. Therefore, by sorting out the development process of national fitness in China, this article understands the opportunities, problems, and requirements facing national fitness at present, and introduces the improved “balanced scorecard” based on policy documents, expert interviews, and relevant literature research. The establishment of the national fitness development index will evaluate the development of China's national fitness in a quantitative form for the first time. According to the index comparison, we can obtain the regional balanced development and growth of national fitness, to help government agencies, business entities, the public, and other stakeholders find the direction and ideas to improve and enhance national fitness.

## Overview of National Fitness in China

### The Development of National Fitness

After China's reform and opening up, the development of national fitness has generally experienced three stages.

#### Full Start-Up Stage (1995–2008)

The relevant policy documents in the full start-up stage are shown in Table 1. In this stage, China's national fitness activities started in full swing, constantly improving the legal system and supporting facilities for national fitness, national fitness activities began to occupy an important position (20).

**Table 1:** Relevant contents of the full start-up stage of national fitness.

**Time**	**Content**	**Issuing subject**
1995.06.20	“Outline of the National Fitness Program”	The State Council
1995.08.29	“Sport Law of the PRC”	The National People's Congress
2002.07.22	“In the opinions on further strengthening and improving the new period sports work”	The State Council
2003.08.01	“Public Cultural and Sports Facilities Ordinance”	The State Council
2004.08.26	“Notice on Further Strengthening the Use and Management of Sports Lottery Public Welfare Fund for National Fitness”	SSAC
2007.05.14	“‘National Fitness and Olympic Games' Series Activities”	SSAC
2008.12.15	“Strive to promote China from a big sports country to a powerful sports country”	Hu Jintao

#### In-depth Development Stage (2009–2014)

The relevant policy documents in the In-depth Development Stage are shown in Table 2. The success of the Beijing Olympic Games not only stimulated the enthusiasm of the people all over the country for sports but also promoted the rapid development of China's national fitness cause. According to statistics, in this stage, China has issued 118 policy documents related to national fitness (21).

**Table 2:** Relevant contents of the in-depth development stage of national fitness.

**Time**	**Content**	**Issuing subject**
2009.10.01	“Regulations on National Fitness”	The State Council
2011.02.15	“National Fitness Program (2011–2015)”	The State Council
2011.04.29	“The 12th Five-Year Plan for the Development of Sports”	SSAC
2012.01.17	“Fifteen years of implementation of the Outline of the National Fitness Program”	SSAC
2013.12.16	“Measures for the Implementation of national Physical Training Standards”	SSAC
2014.01.04	“Opinions on Strengthening and Improving Mass Sports Work”	SSAC
2014.10.20	“The development of the sports industry to promote sports consumption”	The State Council

In this stage, China gradually from a sports country to a sports power, by promoting the development of the sports industry to promote national fitness activities, national fitness has become a national strategy.

#### Optimization and Upgrading Stage (2015–Present)

The relevant policy documents in the Optimization and Upgrading Stage are shown in Table 3. Since 2015, the construction of China's national fitness system has entered a stage of continuous optimization and upgrading.

**Table 3:** Relevant contents of national fitness optimization and upgrading stage.

**Time**	**Content**	**Issuing subject**
2015.11.16	“Remarkable results have been achieved in national fitness activities”	SSAC
2016.05.05	“The 13th Five-Year Plan for Sports Development”	SSAC
2016.06.23	“National Fitness Program (2011-2015)”	The State Council
2016.10.25	“Outline of the ‘Healthy China 2030' ”	The State Council
2017.08.28	“Take meeting people's fitness needs as the starting point of sports work”	Xi Jinping
2019.07.09	“Healthy China Action (2019-2030)”	National Health Commission
2019.08.10	“National fitness is more close to the people, more convenient and more popular”	General Office of the State Council
2019.09.24	“More than 400 million people often take part in physical exercise in China”	“70 years of sports in New China”
2020.10.29	“Proposal of the CPC Central Committee on formulating the 14th Five-Year Plan for National Economic and Social Development and long-range Targets for 2035”	19th CPC Central Committee
2021.08.13	“National Fitness Program (2021-2025)”	The State Council
2021.10.08	“Sports Development Plan of the 14th Five-year Plan”	SSAC

In this stage, the construction of China's national fitness system tends to be improved. The state attaches great importance to national fitness activities, and national fitness has risen to a new height.

### Analysis on Opportunities and Bottlenecks of National Fitness

At present, the development of national fitness is facing major opportunities. First, the development of cross-domain integration has created new opportunities for the development of national fitness, and the development concepts of “integration of sports and health,” “integration of sports and education,” and “combination of sports and tourism” have been gradually deepened. Second, the vigorous development of the social economy has injected impetus into national fitness. The growing diversified fitness needs of the people and the market-oriented operation of non-basic public sports services will promote the prosperity and development of national fitness. Third, the development of modern information technology empowers national fitness. With the rapid development of modern information technologies such as the Internet of things, big data, cloud computing, 5G, and AI, the development of national fitness has been given more possibilities.

The development of national fitness still needs to break through the following problems. First, the overall effect of national fitness needs to be improved. The gap in the development level of mass sports between urban and rural areas and between regions still exists. Second, there is an urgent need to realize the core leading transformation from government satisfaction to people's satisfaction in the performance management of national fitness services. Third, the integrated development ability and effect of national fitness need to be improved.

## Construction of National Fitness Development Index Evaluation System

### Screening and Modification of Index Evaluation Indicators

**Figure 1:**
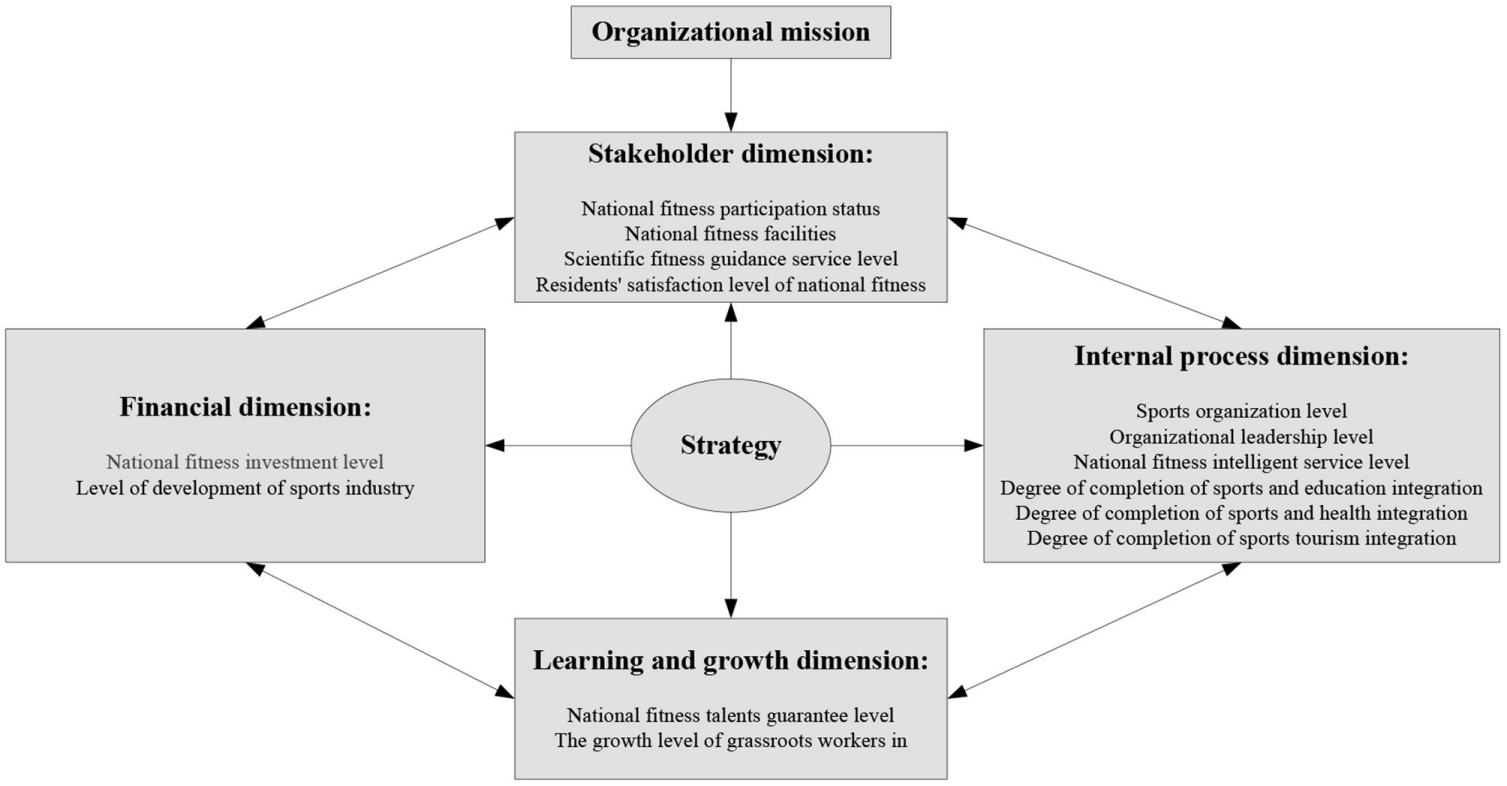
National fitness balanced scorecard model.

This article will introduce the idea of a “balanced scorecard” in the process of constructing the evaluation system of the national fitness development index. The improved “balanced scorecard” method is adopted to set up the index system from stakeholder dimension, financial dimension, learning and growth dimension, and internal process dimension, respectively, to comprehensively reflect the development of national fitness in China (22, 23). National fitness balanced scorecard model is shown in Figure 1.

This study focuses on the development of the four dimensions of national fitness during the 14th Five-year Plan period to ensure the comprehensiveness, consistency, and integrity of the index system. The improved Delphi method was adopted to screen the draft of the national fitness development index, and the final evaluation system was constructed by combining expert subjective judgment with objective data statistics (24). By designing the expert membership questionnaire of the national fitness development index evaluation system, the experts' opinions on the increase, deletion, and merger of indicators were obtained. The reliability and validity of the questionnaires were tested (25–27).

In the process of expert consultation, the questionnaire of “Expert membership degree of national fitness development index evaluation system construction” was distributed to the experts, and the experts were invited to judge the “rationality” of the specific measurement indicators in the evaluation system. “Rationality” refers to the importance of the indicator to the evaluation system. To facilitate quantitative analysis, 1–5 points are used, where 1 means “extremely unreasonable and should be removed,” while 5 means “completely reasonable and should be retained.” The corresponding evaluation results from 1 to 5 are positively increasing. In addition, experts were invited to put forward revised opinions and suggestions on the addition and deletion of measurement indicators. Stata 14 was used for data statistical processing, and the average, standard number, coefficient of variation, and coordination coefficient of each factor layer were calculated. The specific results are shown in Table 4 (28, 29).

**Table 4:** Statistical results of evaluation indicators at all levels.

**Indicators**	**Mean**	**Standard deviation**	**Variable coefficient**
**Factor layer index (*W* = 0.62)**			
Stakeholder dimension	4.62	0.11	0.26
Financial dimension	4.88	0.36	0.38
Learning and growth dimension	4.85	0.33	0.14
Internal process dimension	4.76	0.25	0.31
**The secondary indicators (*W* = 0.53)**			
National fitness participation status	5.00	0.00	0.00
National fitness facilities	4.85	0.38	0.14
Scientific fitness guidance service level	4.77	0.44	0.19
Residents' satisfaction level of national fitness	4.31	0.75	0.56
National fitness investment level	4.38	0.65	0.42
Level of development of sports industry	4.62	0.51	0.26
National fitness talents guarantee level	4.84	0.36	0.11
The growth level of grassroots workers in national fitness programs	3.77	0.73	0.53
Sports organization level	4.38	0.65	0.42
Organizational leadership level	4.69	0.48	0.23
National fitness intelligent service level	4.62	0.51	0.26
National fitness integration development level	3.38	0.87	0.76
**Specific measurement index (*W* = 0.47)**			
Proportion of people in the country who regularly take part in physical exercise	5.00	0.00	0.00
Every 10,000 people have a public sports area	4.92	0.28	0.07
The number of stadiums per 10,000 people	4.84	0.31	0.14
Every ten thousand people have parks, city square area	3.61	0.50	0.27
The number of fitness centers (community sports centers, fitness clubs, etc.) per 10,000 people	4.46	0.52	0.27
School sports facilities and equipment configuration up to standard rate	4.31	0.75	0.56
Opening rate of school sports facilities	4.38	0.77	0.59
Opening rate of public fitness venues	3.77	0.60	0.36
County (city, district) public fitness facilities and community 15-minute fitness circle coverage	5.00	0.00	0.00
Township (street) public fitness facilities and community 15-minute fitness circle coverage	4.69	0.48	0.23
The coverage rate of public fitness facilities and 15-minute fitness circles in administrative villages (communities)	4.07	0.64	0.41
The number of social sports instructors per 10,000 people	5.00	0.00	0.00
The number of sports volunteers per 10,000 people	3.46	0.78	0.60
The number of national fitness monitoring centers per 10,000 people	4.15	0.55	0.31
The proportion of urban and rural residents who have reached the national physical fitness measurement standard	4.85	0.38	0.14
The excellent rate of meeting the national physical and health standards of students	4.53	0.52	0.27
The daily amount of school sports activities for primary and middle school students	4.69	0.48	0.23
The number of extracurricular sports activities for primary and secondary school students every day	3.61	0.65	0.42
Residents' satisfaction with site facilities	4.77	0.44	0.19
Residents' satisfaction with fitness guidance	4.76	0.43	0.18
Residents' satisfaction with fitness activity organization	4.46	0.66	0.42
Per capita investment in fitness facilities	4.62	0.65	0.42
Proportion of sports consumption in per capita disposable income of residents	4.92	0.28	0.08
Per capita investment in fitness activities	3.38	0.87	0.76
Input into training of fitness instructor volunteers	3.46	0.66	0.47
The proportion of public welfare funds invested in sports lottery in national fitness funds	4.62	0.60	0.36
Sports investment accounted for the proportion of total social investment	4.08	0.49	0.24
Proportion of total size of sports industry in GDP	4.92	0.27	0.07
Proportion of sports goods and related products manufacturing in the total size of sports industry	4.23	0.83	0.69
Proportion of sports service industry in the total size of sports industry	4.08	0.64	0.41
The proportion of sports facilities construction in the total size of sports industry	3.63	0.49	0.26
Proportion of added value of competition performance industry	4.31	0.63	0.40
Proportion of added value of fitness leisure industry	4.35	0.52	0.23
Number of sports administrators	3.63	0.68	0.45
Number of sports industry employees	4.48	0.69	0.46
Degree of grass-roots staff involvement in decision-making	4.53	0.51	0.26
Job satisfaction of grass-roots staff	4.42	0.71	0.43
Basic level staff work autonomy degree	4.00	0.71	0.51
Number of training sessions for grass-roots staff	3.52	0.84	0.75
Work reward system for grass-roots staff	3.12	0.82	0.70
Number of grassroots sports organizations	3.38	0.87	0.76
Number of social sports organizations	4.69	0.48	0.23
Number of national fitness clubs	4.07	0.64	0.41
Number of national fitness sites in urban communities	3.77	0.60	0.36
The number of “national sports industry demonstration bases”	3.46	0.56	0.54
Proportion of cooperation between government sports organizations and social sports organizations	3.51	0.76	0.48
The proportion of regular activities carried out by grassroots social sports organizations	3.24	0.89	0.65
Number of joint meetings of national fitness work of people's governments at all levels	4.46	0.52	0.23
The proportion of non-government forces participating in the development of the public service system for national fitness	4.51	0.63	0.41
To track and evaluate the implementation of national fitness and supervise and guide the degree	3.61	0.54	0.25
Number of national fitness information service platforms established at provincial and municipal levels	4.31	0.63	0.39
Number of online and intelligent sports events	4.36	0.77	0.57
Degree of completion of sports and education integration	3.77	0.54	0.34
Degree of completion of body-health integration	3.40	0.73	0.53
Degree of completion of body and travel integration	3.12	0.85	0.79

According to the experts' questionnaire feedback results and the current development of national fitness, the index system is modified (30–32). Finally, combined with many in-depth interviews with experts in the industry, referring to the previous scholars' research on the evaluation system development and combing the related policy documents and leaders' relevant statements (33, 34). After sorting out, the final results of the national fitness development index evaluation system include 4 first-level indicators, 14 second-level indicators, and 49 third-level indicators, as shown in Table 5.

**Table 5:** Final results of national fitness development index evaluation system.

**Level indicators**	**Secondary indicators**	**Tertiary indicators**
Stakeholder dimension (A)	National fitness participation status (A1)	Proportion of people in the country who regularly take part in physical exercise (A11)
	National fitness facilities (A2)	Every 10,000 people have a public sports area (A21)
		The number of stadiums per 10,000 people (A22)
		The number of fitness centers (community sports centers, fitness clubs, etc.) per 10,000 people (A23)
		School sports facilities and equipment configuration up to standard rate (A24)
		County (city, district) public fitness facilities and community 15-minute fitness circle coverage (A25)
		Township (street) public fitness facilities and community 15-minute fitness circle coverage (A26)
		The coverage rate of public fitness facilities and 15-minute fitness circles in administrative villages (communities) (A27)
	Scientific fitness guidance service	The number of social sports instructors per 10,000 people (A31)
	level (A3)	The number of sports volunteers per 10,000 people (A32)
		The number of national fitness monitoring centers per 10,000 people (A33)
		The proportion of urban and rural residents who have reached the national physical fitness measurement standard (A34)
		The excellent rate of meeting the national physical and health standards of students (A35)
		The daily amount of school sports activities for primary and middle school students (A36)
		The number of extracurricular sports activities for primary and secondary school students every day (A37)
	Residents' satisfaction level of	Residents' satisfaction with site facilities (A41)
	national fitness (A4)	Residents' satisfaction with fitness guidance (A42)
		Residents' satisfaction with fitness activity organization (A43)
Financial dimension (B)	National fitness investment level (B1)	The total size of residents' sports consumption (B11)
		Per capita investment in fitness activities (B12)
		Sports lottery public welfare fund input (B13)
		Total financing of sports industry (B14)
	Level of development of sports	Total size of sports industry (B21)
	industry (B2)	The proportion of added value of sports industry in GDP (B22)
		Sports goods and related products manufacturing added value (B23)
		Added value of sports service industry (B24)
		Added value of sports facilities construction industry (B25)
		Proportion of added value of competition performance industry (B26)
		Proportion of added value of fitness leisure industry (B27)
Learning and growth	National fitness talents guarantee	Sports industry legal entity (C11)
dimension (C)	level (C1)	Number of sports industry employees (C12)
	The growth level of grassroots	Degree of grass-roots staff involvement in decision-making (C21)
	workers in (C2)	Job satisfaction of grass-roots staff (C22)
		Basic level staff work autonomy degree (C23)
Internal process	Sports organization level (D1)	Number of social sports organizations (D11)
dimension (D)		Number of national fitness sites in urban communities (D12)
		The number of “national sports industry demonstration bases” (D13)
		Proportion of cooperation between government sports organizations and social sports organizations (D14)
		The proportion of regular activities carried out by grassroots social sports organizations (D15)
	Organizational leadership level (D2)	Number of joint meetings of national fitness work of people's governments at all levels (D21)
		The proportion of non-government forces participating in the development of the public service system for national fitness (D22)
	National fitness intelligent service level (D3)	Number of national fitness information service platforms established at provincial and municipal levels (D31)
		Number of online and intelligent sports events at the national level (D32)
	Degree of completion of sports and	Number of sports traditional characteristic schools (D41)
	education integration (D4)	Number of national youth sports clubs (D42)
	Degree of completion of sports and	Number of sports rehabilitation centers (D51)
	health integration (D5)	Number of sports therapists (D52)
	Degree of completion of sports	National Sports Tourism Demonstration base (D61)
	tourism integration (D6)	Market size of sports tourism industry(D62)

### National Fitness Development Index Evaluation Index Meaning

#### Stakeholder Dimension

The stakeholder dimension includes four secondary indicators, namely, participation status of national fitness, facilities supply of national fitness venues, scientific fitness guidance service level, and residents' satisfaction level of national fitness conditions.

The proportion of people who regularly take part in physical exercise is used to reflect the state of national fitness participation. Regular physical activity was defined as more than three times per week of moderate-intensity exercise lasting more than 30 min.

National fitness facilities include sports venues, fitness centers, school sports facilities, etc. The area of public sports venues per 10,000 people can reflect the scale of sports venues and facilities. The standard rate of school stadium facilities and equipment is the necessary condition and material guarantee to carry out normal physical education. The coverage rate of public fitness facilities and 15-min fitness circles in counties (cities, districts), towns (streets), and administrative villages (communities) can reflect the regional distribution of national fitness venues and ensure the demand of residents for fitness activities.

To improve the service level of scientific fitness guidance, it is related to the number of social sports instructors, sports volunteers, and national physical monitoring centers. Social sports instructors are those who provide voluntary service for national fitness to the public and obtain technical ratings. The National Fitness Monitoring Center is a non-profit organization engaged in various kinds of national fitness testing and provides the scientific basis for promoting the implementation of the national fitness plan. And “national student physical health standard” is specially used to evaluate the health status of students in school standards. Young people's sports activities are an important way to promote the healthy growth of young people. The participation of youth in national fitness was measured by the daily physical activity hours of primary and secondary school students in and out of school.

In addition, citizens' satisfaction with national fitness is mainly reflected in three aspects: venue facilities, fitness guidance, and activity organization.

#### Financial Dimension

The financial dimension of national fitness is reflected in the input level and output level of national fitness (the development level of national fitness).

The total scale of residents' sports consumption includes the service and life consumption of residents' national fitness, which can reflect whether the current national fitness service can meet the demand. To a certain extent, the per capita investment in fitness activities reflects the government's emphasis on national fitness. The public welfare fund of sports lottery is the main source of funds for fitness activities, which can support the construction of a large number of public service venues for national fitness and the holding of mass sports activities.

The output dimension of national fitness is mainly reflected by the current development level of national fitness. The industrial scale is an important index to measure the development of national fitness. The reasonable industrial structure is of great significance to promote the high-quality development of the national fitness industry. The added value of the sports industry includes the added value of sports goods and related products manufacturing, the added value of sports facilities construction, and added value of sports services. Among them, fitness, leisure, and competition performance are the core of the sports service industry and the main direction of national fitness industry development in the future.

#### Dimension of Learning and Growth

The dimensions of learning and growth of national fitness include the level of talent guarantee and the level of growth of grass-roots staff.

The number of employees and the number of industrial legal entities, respectively, reflect the capacity of the industry to absorb the employment population and the power of market subjects. The future development of national fitness is closely related to the participation of grassroots staff.

The level of grassroots staff's participation in decision-making, job satisfaction, and degree of autonomy can largely determine the promotion and implementation of national fitness among the masses.

#### Internal Process Dimension

The internal process dimension of national fitness represents the development capability of the national fitness industry, including the level of a sports organization, organization leadership, intelligent service, and integration with education, health, and tourism.

Social sports organizations refer to non-profit social organizations that carry out nationwide fitness and sports competition activities and improve the fitness level of the masses. Grassroots social sports organizations include government sports organizations and social sports organizations, and their participation ratio can show the current marketization level of national fitness. The national sports industry demonstration base is the starting point of the top-level design of national sports industry development and the benchmark of regional sports development.

The level of organization and leadership is reflected in the number of joint meetings of the national fitness work of people's governments at all levels and the proportion of the government absorbing social forces to participate in the national fitness construction work. The National Fitness Plan (2021–2025) points out that the national fitness service level should be promoted by establishing provincial and municipal information service platforms and carrying out online and intelligent sports events.

The integrated development of the national fitness industry includes the integration of physical education, physical health, and sports travel. The number of traditional sports characteristic schools and the number of national youth sports clubs can directly reflect the current development of sports and education integration. The transformation from sports medical integration to sports health integration, through the establishment of sports rehabilitation centers and the training of sports rehabilitation teachers to provide sports rehabilitation plans for patients with chronic diseases and improve the sports rehabilitation level of the whole society. Sports is an important resource to develop the tourism industry, and tourism is an important driving force to promote the sports industry. The number of national sports tourism demonstration bases and the market size of the sports tourism industry are adopted to represent the level of sports tourism integration.

## Empirical Study

This article calculates the weight of each index through the analytic hierarchy process (AHP) by aiming at the index system of national fitness development. The corresponding data of the last 5 years were collected as the base data of the index system, and the target value of each index needed to be achieved in the end, and the interval reference standard of the national fitness evaluation system was established, to form a complete national fitness development index evaluation system. The evaluation system can be used to calculate the development index in the next 5 years, which can be used as a tool to measure the development of national fitness in China in the future.

### Weight Calculation of National Fitness Development Index

**Figure 2:**
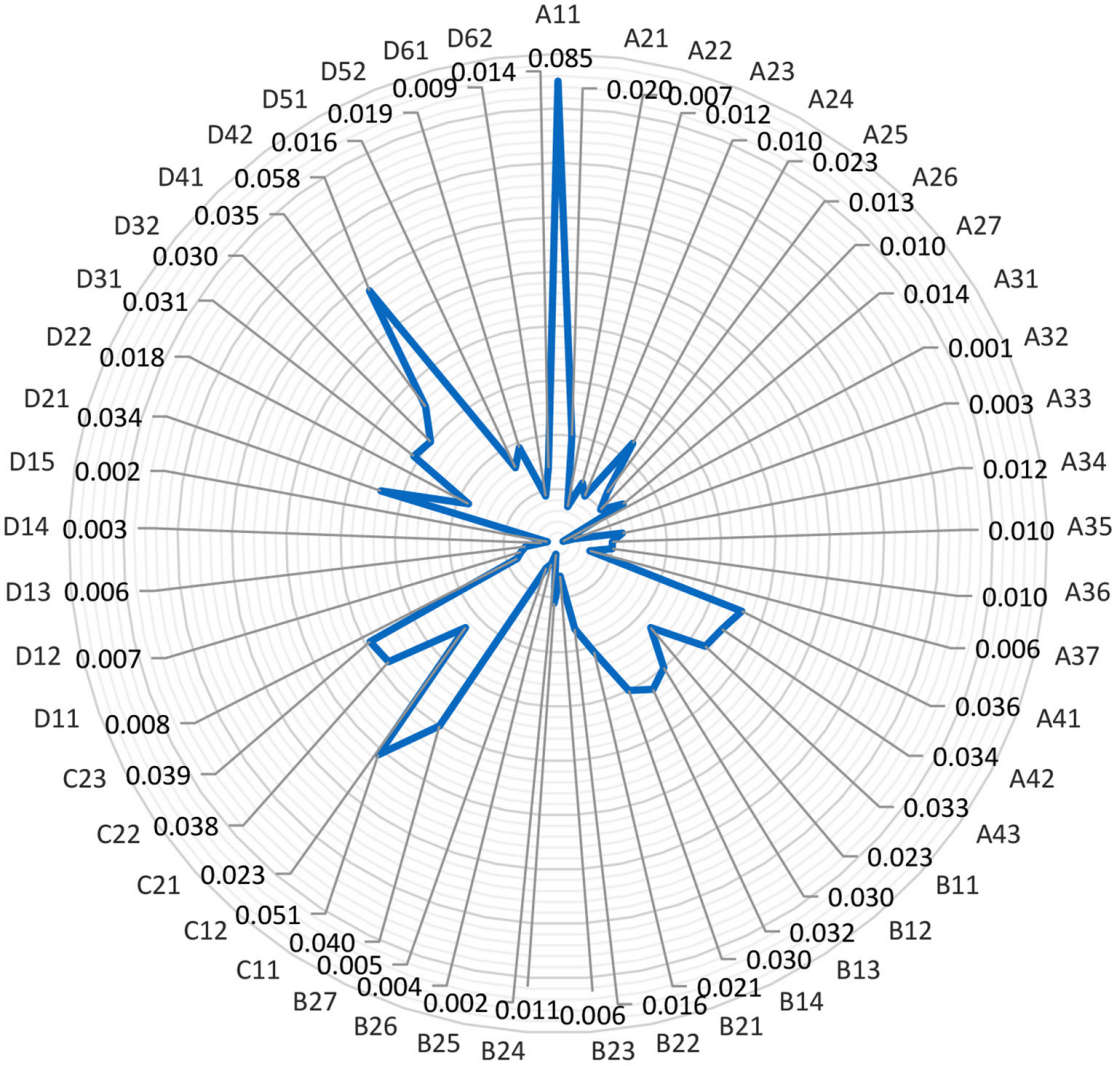
The evaluation system of the national fitness development index and the weight value of each evaluation index.

The reasonable degree of weight setting will affect the objectivity and fairness of the results. In this study, experts were invited to score the weights, AHP was used to distinguish the weights of evaluation indexes, a hierarchical model was established according to the evaluation index system, and a judgment matrix was constructed. Finally, the corresponding weight coefficients of evaluation indexes at each level were obtained (Figure 2).

### Interval Reference Standard of National Fitness Development Index Evaluation System

**Figure 3:**
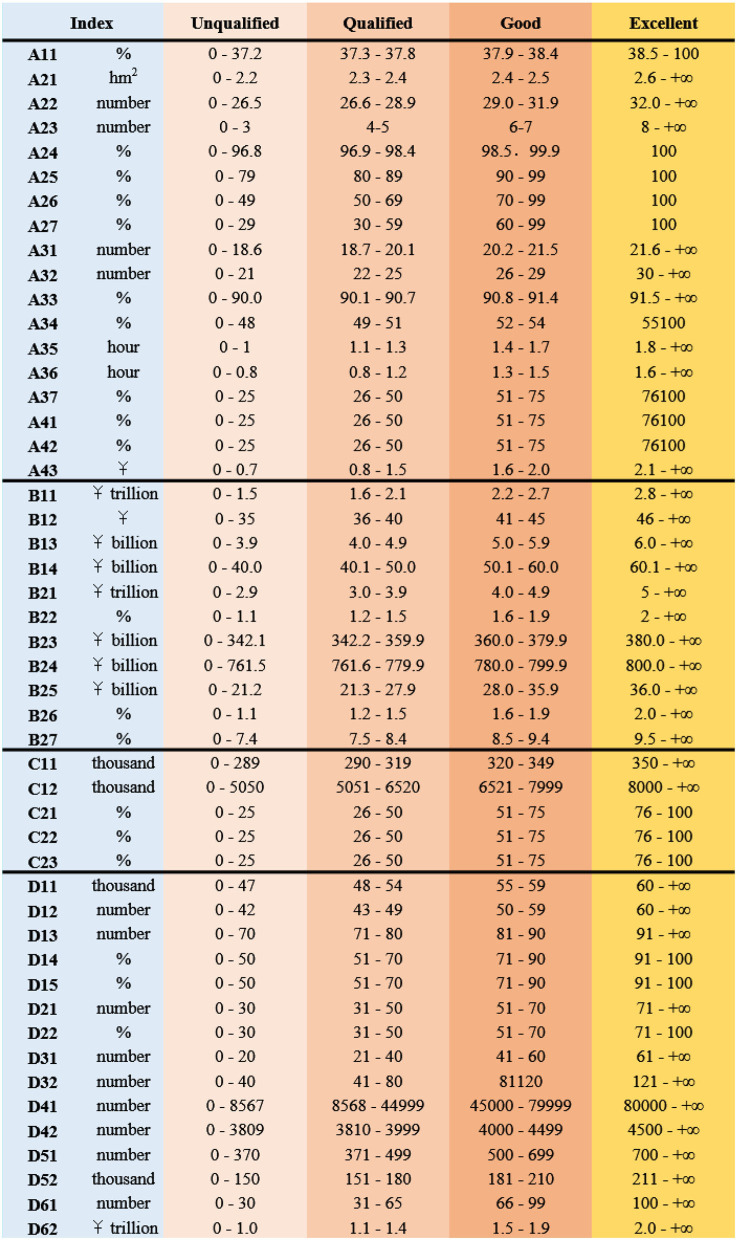
National fitness assessment index interval reference standard table.

Taking the relevant data of the last 5 years as the base period data, the target value method is adopted to set the target data. In the evaluation of the national fitness development index, the index less than the base period is unqualified, the index greater than the target data is excellent, and the index between the two is qualified and good, respectively. Points 25, 50, 75, and 100 are awarded to unsatisfactory, acceptable, good, and excellent, respectively. The development of national fitness is a relative and dynamic stage process, so the development index is evaluated by selecting the goal-status evaluation method. National Fitness assessment index interval reference standard is shown in Figure 3. The symbol in Figure 3 represents the evaluation index, which has been explained in Table 5.

For quantitative indicators, benchmarking method and local fitting method are, respectively, used to set standards. The benchmarking method is based directly on the target values of relevant national plans, programs, and opinions. Among them, the “14th Five-year Plan for Sports Development” and “National Fitness Plan (2021–2025)” are programmatic documents for national fitness and the most important basis for establishing a comprehensive evaluation system. Partial fitting method: for some indicators with no clear target value, collect official statistical data, functional department work reports, consult relevant personnel, count relevant data in the past, and calculate the target value of the required variable according to the growth rate.

The achievement evaluation of qualitative indicators is mainly based on the objectives and tasks in relevant documents and plans, and the rationality of the achievement standard of each evaluation indicator is scored by experts.

Finally, the national fitness development index was established, including 4 first-level indicators, 14 second-level indicators, and 49 third-level indicators. It can calculate the national fitness development index with a total score of 100 points. Through index calculation, it can help government agencies, enterprises, the public, and other stakeholders to know the current development status and improvement direction of national fitness.

### Result Analysis

Through the calculation of the weight of national fitness development indicators and the formulation of interval reference standards, it can be seen that the current development of national fitness should focus on the following areas:

Based on system construction, the legal environment for the implementation of the national strategy of national fitness is improved. The legislation and revision of laws and regulations in key areas of national fitness are strengthened, and a policy guarantee for the construction of a national fitness development index evaluation system is provided.

With resource integration as the core, the construction of a new pattern of coordinated governance of national fitness is deepened. The integrated development of national fitness is the general trend of the development of the times. The integration of national fitness with health, education, culture, science and technology, tourism, pension, and other fields is strengthened.

The main contradiction is taken as the starting point to make up for the shortboard of public service supply of national fitness. We should pay special attention to making up the shortcomings from the three aspects of the supply of national fitness venues and facilities, the supply of events and activities, and the supply of guidance services, to promote the equal development of public services for national fitness.

With the goal of enabling science and technology, the construction level of intelligent fitness services for the whole people is improved. The construction of an intelligent information platform for national fitness service supply is promoted, and the research on key technologies and products in the intelligent field of national fitness is strengthened.

## Conclusion

At present, China is in the key stage of transforming into a “sports power,” with both opportunities and challenges. How to grasp this key journey, build a higher level and higher quality national fitness, and form a national fitness work pattern of “government led, social coordination, public participation, and legal guarantee” is an important topic. Based on a deep understanding of the important connotation of “national fitness under social change,” this article uses the improved “balanced scorecard” method to construct an evaluation index system of national fitness development, including 4 primary indicators, 14 secondary indicators, and 49 tertiary indicators. Through the combination of qualitative analysis and quantitative analysis, the interval reference standard of the national fitness evaluation system is evaluated, and finally, the national fitness development index evaluation system is formed, to provide evaluation tools and theoretical reference for the development and improvement direction of national fitness.

## Data Availability Statement

The original contributions presented in the study are included in the article/supplementary material, further inquiries can be directed to the corresponding author/s.

## Author Contributions

ZL and LL: conceptualization and methodology. ZL and YZ: resources. ZL and BH: supervision. ZL, BH, and SZ: writing—original draft. SZ: validation, software, and formal analysis. LL, YZ, RL, BH, and SZ: investigation. RL: visualization. ZZ and BH: data curation. YZ, RL, SZ, and ZZ: writing—review and editing. All authors contributed to the article and approved the submitted version.

## Funding

This study was supported by the National Natural Science Foundation of China (Grant No. 71901141), the Soft Science Research Project of Shanghai Science and Technology Committee (Grant No. 22692104000), Municipal Key Curriculum Construction Project of University in Shanghai (Grant No. S202003002), and Shanghai Philosophy and Social Science Planning Project (Grant No. 2020BGL007).

## Conflict of Interest

The authors declare that the research was conducted in the absence of any commercial or financial relationships that could be construed as a potential conflict of interest.

## Publisher's Note

All claims expressed in this article are solely those of the authors and do not necessarily represent those of their affiliated organizations, or those of the publisher, the editors and the reviewers. Any product that may be evaluated in this article, or claim that may be made by its manufacturer, is not guaranteed or endorsed by the publisher.
